# The geopolitical turn in biotechnology: Implications for Science and Policy

**DOI:** 10.1038/s44319-025-00658-4

**Published:** 2025-12-04

**Authors:** Ruth Mampuys, Haroon Sheikh

**Affiliations:** 1 Netherlands Council for Government Policy (WRR), The Hague, The Netherlands; 2 Department Theory and Methodology, Erasmus School of Law Rotterdam, Rotterdam, The Netherlands; 3 Faculty of Politics, Philosophy and Economics, Vrije Universiteit Amsterdam, Amsterdam, The Netherlands

**Keywords:** Biotechnology & Synthetic Biology, Economics, Law & Politics, Science Policy & Publishing

## Abstract

Biotechnology is increasingly being regarded as a national strategic and security asset, rather than as scientific achievement and solution to broader global challenges. This shift towards techno-nationalism has significant positive and negative implications for scientists, businesses and policy makers with long-term consequences and risks.

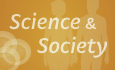

For decades, biotechnology has featured prominently in numerous foresight reports, often with high expectations regarding its transformative potential in agriculture, medicine, and industry. The emerging new characterization of biotechnology as a “strategic” or “security” asset rather than as a scientific field and potential solution to broader global challenges marks therefore a significant shift. Global technological competition is certainly not a new phenomenon (Capri, 2025). But the stakes are significantly higher in the current geopolitical dynamics of increasing tensions and conflicts.

The impact of this shift is amplified by several other factors. First, the erosion of an imperfect yet relatively stable international order, alongside a weakening of multilateral institutions, potentially destabilizes international agreements to address risks, weaponization, and ethics. Second, the rapid convergence of emerging technologies such as artificial intelligence (AI) with biotechnology has accelerated developments and increased international rivalry. Finally, due to decades of globalization and convergence, the impact of this shift will be both broader and deeper, affecting professionals and policymakers throughout the world. An increasing number of domains are being drawn into the geopolitical arena, and technological leadership is no longer merely a matter of prestige—it is becoming a matter of national survival (Mampuys et al, 2025). Biotechnology is no exception.

“**…** the erosion of an imperfect yet relatively stable international order, alongside with a weakening of multilateral institutions potentially destabilizes international agreements to address risks, weaponization and ethics.”

A characteristic of the geopolitical developments of recent decades is the expanding range of domains through which power is exercised (Galeotti, 2022). This article illustrates how biotechnology is increasingly being regarded as a national strategic and security asset, rather than as a scientific achievement and part of a solution to global challenges. That shift may, in the short as well as long-term, have significant implications for scientists, businesses, and policymakers. While the benefits of this increased interest and rise of investments should be harnessed where possible, it is of critical importance that long-term consequences and risks—such as effects on academic freedom, international cooperation, privacy, biodiversity or genetic surveillance—are not overlooked in the decision-making processes that guide future directions.

“**…** biotechnology is increasingly being regarded as a national strategic and security asset, rather than as scientific achievement and part of a solution to global challenges.”

## From technological trend to geostrategic asset

Biotechnology is increasingly regarded not merely as a research field or a catalyst for innovation in sustainability and public health, but as a geopolitical instrument, one that can be leveraged for military and economic advantage to compete with rival states.

The USA has been a global frontrunner in biotechnology for a long time, but the policy tone has changed rapidly over the past years. In April 2025, the National Security Commission on Emerging Biotechnology (NSCEB) issued its recommendations to Congress. Chair and Senator Todd Young said in the press release: “it [biotechnology] is no longer constrained to the realm of scientific achievement. It is now an imperative for national security, economic power, and global influence.” The prioritization of national security and increasing competition with China is moreover mentioned in US publications as a key driver for changes in strategy, since the latter has over the years steadily caught up or even surpassed the USA in some areas (see also the ASPI technology tracker).

Biotechnology has also been central to China’s science modernization since the mid-2000s with industrial policy, genome sequencing, AI integration and strategic aims to surpass the USA and Europe. Here too, a transition can be seen from “regular” technological/scientific competition towards an increased focus on strategic autonomy and security. China’s 2015 *Made in China 2025* plan first articulated a framework for technological competitiveness, reinforced during the early COVID-19 period. In 2022, a five-year bioeconomy plan sought to achieve *independence* and *scientific self-reliance*, and later that same year, biosecurity was designated a pillar of Xi Jinping’s *comprehensive national security framework*. In contrast, earlier five-year plans referred to biotechnology mostly in the context of ‘international exchange and cooperation’ (2011 – 2015), and to develop ‘globally competitive biotechnology companies to achieve biotech breakthroughs’ (2016 – 2020).

Both China and the USA justify the shift toward securitization and the corresponding investments by invoking the risk of technological misuse by the other state—such as development of bioweapons or specific ethnic genetic attacks—an argument characteristic of the technological arms races observed throughout history, such as the “Space Race”.

“Both China and the USA justify the shift toward securitization and the corresponding investments by invoking the risk of technological misuse by the other state…”

The EU has also awakened to the increasing importance of technology as a strategic asset. The 2024 Draghi report explicitly placed European technology and competitiveness in the frame of geopolitical rivalry. In 2025, the European Commission published a new life sciences strategy and announced a comprehensive 'Biotech Act” by 2026. The European approach seems to be still rooted in an ambition for innovation and scientific excellence, but here too, the language also hints at geopolitical motivations: “*biotechnology and biomanufacturing can help enhance the EU’s strategic autonomy, resilience and circularity” […]* and “*reduce dependence on non-EU actors” […] and the EU seeks “strategic partnerships with reliable global actors*”.

While the EU pursues *strategic autonomy*, Japan advances the concept of *strategic indispensability* and has appointed a minister of economic security to coordinate efforts and protect the nation’s position in critical technological sectors under growing external pressure. Reflecting this approach, the updated bioeconomy strategy of 2024 responds to “intensifying international competitions” by focusing investments on areas where Japan aims to be globally indispensable. With regard to biotechnology, South Korea promotes *bio-sovereignty*. Its 2022 *Digital Biotechnology Innovation Strategy* aims to enhance technological competitiveness and self-reliance amid global competition, explicitly citing the USA and China as competitors. In early 2025, a *National Bio Committee* was installed to coordinate biotechnology policy, accelerate innovation and advance national *biotechnology sovereignty*. A last example is NATO’s *International strategy on biotechnology and human enhancement technologies* (2024) which emphasizes the responsible development and use of these technologies in security and defense applications across the alliance.

## Changing balance of key policy interests

The implementation of these strategies likely has profound implications for scientists, businesses and policymakers. At a more conceptual level, this strategic shift can be understood as a change of policy priorities—from an emphasis on prosperity and values towards security and resilience.

Policy development typically requires balancing three core interests: security, prosperity, and values. Policies are generally designed to avoid compromising security/safety and where possible to enhance it; they ideally contribute to prosperity while safeguarding fundamental societal values. These elements are closely interlinked and cannot easily be considered in isolation. To prosper, a secure and stable environment is needed, while security also requires monetary, material, and human investments. In terms of broad welfare, a secure and prosperous society can only exist if it also upholds individual and collective values on which that society is built.

In biotechnology policy development, security, prosperity, and values also play a key role. The BWC (1972), Asilomar conference (1975) and The Cartagena Protocol on Biosafety (2003) led to national regulations and policies aimed at managing physical and security-related risks associated with biotechnology. There have also been high expectations for biotechnology to deliver prosperity through improved food production, the creation of novel therapies, and creating a bioeconomy that is both profitable and sustainable. Finally, values have always played a significant role, focusing on both moral, ethical or religious concerns as well as positive values, such as improving health, equity, sustainability, and so on.

“In biotechnology policy development, security, prosperity and values also play a key role.”

While the specific interpretation and prioritization of these three elements may vary across countries and regions, the ongoing geopolitical conflicts are likely to exacerbate tensions between them (Mampuys et al, 2025). After decades of relative stability and peace, geopolitical conflicts are on the rise again and in a much broader arena than before, when they were focused predominantly on the military. Multilateral institutions are under pressure, and many countries have started to increase spending on defense and prioritize autonomy and sovereignty. This has significant implications for the balancing of key policy interests, resulting in a reorientation towards security and resilience, potentially at a cost of prosperity and values.

## Policy instruments to promote, protect, and partner

The impact of this reorientation from scientific advancement to geostrategic asset—and from prosperity and values towards security—has been noticeable for several years. The promote–protect–partner distinction, which the EU also employed in its 2024 Economic Security Strategy, provides a useful framework for looking at these approaches, which can be observed in many technological fields such as semiconductors, quantum computing, medical technology, aerospace, and biotechnology.

Countries are expanding funding to promote specific areas of biotechnology as part of their national strategies. The EU, China, Japan, India, USA, South Korea all developed plans for leading the biobased economy. Optimistic outlooks predict that more than 60% of physical inputs in the global economy could be produced or substituted through biomanufacturing in 2040, contributing to countries’ strategic autonomy as it potentially decreases dependencies on countries that are (future) rivals or adversaries. Another field that sees an increase in funding is defense and security-related research, including dual-use. In the biotech domain, these investments focus on research on pathogens and medical countermeasures (MCM) in the perspective of bioweapons but also on the development of innovative materials and human enhancement research. China’s Peoples Liberation Army (PLA) has shown an increasing interest in biotechnology to expand its military capabilities, as well as the USA and the NATO alliance (https://www.fdd.org/analysis/2025/01/15/biotech-battlefield/; https://www.rand.org/pubs/commentary/2022/10/biotechnology-and-todays-warfighter.html). The EU adopted a white paper in 2024 that explores dual-use technologies, followed by two expert reports in 2025 explicitly addressing the potential of biotechnology in this regard. Japan is pushing to build a “dual-use startup ecosystem” while South Korea announced a “K-defense startup strategy”.

Meanwhile, measures are taken to protect national markets from international competition and exploitation. Instruments like foreign investment screening tools—such as FDI in Europe, the Committee on Foreign Investment (CFIUS), or the entities lists in the USA—serve to protect local knowledge and data. Both the USA and China are in the process of enacting legal measures to protect their citizens’ Digital Sequence Information (DSI) from access by specific state and non-state actors (https://www.justice.gov/opa/pr/justice-department-implements-critical-national-security-program-protect-americans-sensitive; https://www.science.org/content/article/congress-close-passing-law-would-freeze-out-certain-chinese-biotechs). While security reasons are the primary argument given for these restrictions, the vast amount of data gathered during the last decades is also a potential goldmine for developing the bioeconomy in light of recent advances in AI (Puglisi and Rask, 2024).

Policy instruments such as tariffs or export bans to curb or even prohibit the activities of foreign actors are mainly deployed by the major geopolitical powers, but indirectly affect many other countries as well. The trade war between the USA and China affects biotechnology R&D directly through tariffs on products such as precision instruments from the USA or lab reagents and glassware from China. Furthermore, activities, such as clinical trials, are restricted citing intellectual property theft risks and violations of human rights.

The proposed US *Biosecure Act* would prohibit so-called ‘biotechnology companies of concern’ tied to foreign adversaries like China, Russia, Iran, and North Korea to operate on the US market under the flag of security risks. Restricting access to funds is another example. EU member states have proposed to restrict eligibility for dual-use and defense projects in the Horizon Europe program to a limited set of countries. Vice versa, China also began tightening its biosecurity by introducing a dedicated law in 2020, with stricter management of human genetic resources and restrictions for foreign pharmaceutical and biotechnology firms operating within its borders. The Anti-Foreign Sanctions Law from 2021 enables retaliation against countries that impose sanctions, affecting foreign access to Chinese biotech. To protect local industries against tariffs, national support packages may be created, such as in the biopharmaceutical sector in South Korea.

This “lawfare”, weaponization of regulations, restrictions, and controls, has implications for regulatory complexity and compliance burdens, potentially resulting in supply chain disruptions, corporate restructuring, and reactions by financial and equity markets. Given the globalized markets and R&D environments, these effects extend far beyond the Chinese and US markets but affect many countries active in biotechnology.

“This ‘lawfare’, weaponization of regulations, restrictions and controls, has implications for regulatory complexity and compliance burdens…”

Finally, the search for new partnerships and markets underscores the geopolitical shift. A notable example is China’s *Belt and Road Initiative* and its engagement with diagnostic laboratories and vaccine diplomacy in the Global South during the COVID-19 pandemic. Years later, the EU launched its *Global Gateway Initiative* and announced plans to pursue further international biotech and biomanufacturing partnerships to foster *strategic cooperation* and *diversify global supply chains*. Other examples are the recent US partnerships with Japan and South Korea on AI, quantum computing, and biotech, the Helsinki Global Biosecurity Accelerator (GBA), or the 2024 Seoul and Tokyo biosecurity dialogs with the Council on Strategic Risks (CSR). These new partnerships often go beyond supply chain diversification and dependency management; they also aim to build strategic influence by setting standards and shaping agendas. While standard-setting yields economic benefits, establishing a common discourse can enhance leverage in multilateral institutions, such as UN agencies.

## Implications for science, business, and policy

We discuss three types of implications of these promote–protect–partner approaches: winners, losers, and risks; conflicting rationalities and long-term-costs of an unbalance between security, prosperity, and values.

### Winners, losers, and risks

Increasing investments or regulatory easing for national actors may serve as positive incentives. Simplifying and/or easing regulations can lower the barrier for R&D and startups, and more funding can give the field a boost to reach the potential it has been associated with for many years. However, these changes also carry risks and downsides. First, these investments probably do not end up evenly spread out. More likely, they end up in specific domains and with specific actors. Priority fields are focused on increasing wealth and resilience through biomanufacturing of critical materials—fuels, plastics, food derivatives, vitamins and so on—emergency-responsive health care and defense applications. Available funds are also prone to target ambitious startups and entrepreneurs in countries with a well-functioning regulatory system and high-ranking universities and research institutes. Fields and actors with less commercial potential, such as fundamental research, rare-diseases or sustainability, or with limited market access, for instance, low and middle-income countries, will likely lag behind. As such, these dynamics risk to contribute to the further marginalization of the Global South and funding for climate/sustainability research.

Second, geopolitically-driven policy instruments may contribute to the forming of new blocs and to a decline in openness and transparency. Increasing competitiveness and security priorities encourage the formation of new partnerships and the development of independent systems and standards (Saric et al, 2025). Such emerging fragmentation may complicate knowledge-sharing and cooperation between scientists and other biotech professionals.

“**…** geopolitically-driven policy instruments may contribute to the forming of new blocs and to a decline in openness and transparency.”

Third, more funding for dual-use research risks putting scientists in a more difficult position to execute research, engage in international collaboration, and ensure compliance with growing export controls and security measures, particularly since the boundaries between military and civil-purpose research are often not clear.

The tech rivalry, grand ambitions, and hurried nature of technological development have been identified as a risky cocktail that might lead to unintended consequences, whether it is an accident triggering a new pandemic or a genetic arms race (Biberman, 2024). The BWC, in which global agreements have been made not to develop, produce, or stockpile biological and toxin weapons, has been confronted with enforcement challenges from its initiation. An increase in dual-use research and increasing rivalry in the defense domain will only enlarge these challenges, putting crucial international agreements like these under further pressure.

“The tech rivalry, grand ambitions and hurried nature of technological development have been identified as a risky cocktail that might lead to unintended consequences…”

### A world of conflicting rationalities

The geopoliticisation of biotechnology will challenge scientists, businesses, and policymakers to navigate a different rationale than they are used to. The logic of geopolitics conflicts in several ways with the world they are familiar with (Mampuys et al, 2025).

A first clash of rationalities emerges between academic freedom and knowledge exchange and the realities shaping international collaborations. Examples are concerns about cooperation with Chinese universities with alleged ties to the PLA (Hannas and Tatlow, 2020), or pressure on universities’ collaborations with Israeli universities. In Europe, recent geopolitical events have shaped an evolution from “open innovation, open science, open to the world” (2015) to “as open as possible and as closed as necessary” (2020) (Saric et al, 2025). Several countries and regions—such as the EU, the USA, and Australia—are in the process of developing and implementing screening mechanisms for international students. While these measures are aimed at preventing security risks, they also put educational institutions in a difficult position and may over time limit the international flow of talented professionals.

A second clash arises between geopolitical imperatives and free-market principles, affecting a wide range of technology sectors, including biotechnology. Government interventions—such as the US Biosecure Act or measures related to the global chip competition—aimed at securing critical resources, skills or capabilities, often conflict with liberal market norms and World Trade Organization (WTO) principles. Historically permitted as WTO exceptions, the protection and restriction of information and technologies under the pretext of national security is increasingly problematic, fostering a growing gray zone between genuine security measures and economic protectionism.

A third example of conflicting rationalities is an increase in high-risk research that states have long sought to prevent (others from doing) or limit in scale and scope. While not new, this contradiction has gained renewed significance by calls for more dual-use and specific defense-purpose research. Breakthrough developments, such as human genome editing, gene drives or mirror life, have always been approached with significant caution, strict safety and security protocols, sometimes even leading to (temporary and/or national) moratoria. Increasing geopolitical tensions risk a more hurried decision-making into engaging in such research with a prioritization of competition instead of caution. In this light, several authors have already called for strengthening the agreements and enforcement options of the BWC.

### The long-term costs of prioritizing national security

Prioritizing national security and resilience in biotechnology challenges the balancing act of core policy priorities, with potential long-term costs on fundamental values. In the pursuit of greater geopolitical resilience and competitive power, there appears to be a growing willingness to engage in high-risk research framed under biosafety and biosecurity imperatives. This development may lead to downplaying or sidelining safety concerns out of fear of falling behind. The logic of “if we do not do it, they will” may drive states into a dangerous spiral, illustrated by the nuclear arms race (Mearsheimer, 2001). The potential risks of applications of gain-of-function (GoF) research, human genome editing, gene drives or mirror life are significant, in some cases irreversible once out of the lab. The precautionary or “look before you leap” approach (Evans et al, 2025) that has characterized biotechnology regulation for a long time, may be put under serious pressure in the increasing rivalry between top countries.

A less dramatic but nevertheless serious example is the control and use of DSI for innovations serving national interests. While this may advance resilience or even public health goals, it can simultaneously undermine individual autonomy, informed consent, and privacy. In addition to geopolitical risks posed by foreign actors and adversaries, domestic risks loom when DSI databases are over time repurposed for surveillance, discrimination or political repression. ‘Mission creep’ is a well-known effect after crisis situations, where the status quo does not return once the crisis is over, and some aspects remain, such as security measures after 9/11 or COVID-19. Genetic surveillance misuse is already evident in China, where authorities use genetic data for surveillance and repression of ethnic minorities (Byler, 2021). Even less severe uses—such as in (health) insurance or travel restrictions—pose significant human rights concerns. This raises urgent questions about governance, accountability and the ethical boundaries of state use of biological data.

Over the years, vast amounts of DSI have been collected and stored without adequate consideration for security or privacy safeguards in view of the current technological capabilities. International debate on the use and protection of DSI are on the rise, but adequate global governance remains fragmented (Mampuys and Cruz, 2026). Increasing geopolitical rivalry and data hunger of AI models may also lead to increasing data scraping or biopiracy for non-human genetic resources. This puts middle and lower-income countries at a disadvantage by depriving them of opportunities for innovation, and will further undermine the enforcement of international agreements such as the Nagoya Protocol.

## Conclusion

The geopolitical dynamics in the world present both risks and opportunities for biotechnology. Prioritizing and investing in the sector can stimulate innovation and shift—legal or even ethical— boundaries in ways that may lead to major breakthroughs. On the other hand, these shifts may also carry undesirable consequences, such as downplaying risks to human health and the environment, creating obstacles to knowledge-sharing, violating genetic privacy, and deepening divides between countries and geopolitical blocs. While significant technological challenges remain, we should not underestimate the potential impact current radical technology and policy changes can have.

There are no simple solutions to mitigate the potential negative implications of the geopolitical dynamics, but as a start it is important to both recognize and acknowledge that fundamental choices are being made that may have long-term and, in some cases, irreversible consequences. Major powers play a key role in setting the tone and/or exercising caution with this promising yet high-risk technology, which calls for a deep and sustained form of diplomatic engagement (Biberman, 2024). Meanwhile, professionals in the field also have a responsibility to use their expertise and experience to make the implications of these choices explicit to policymakers, enabling scientifically informed policy decisions that balance security, values, and prosperity in both the short- and long-term.

“Meanwhile, professionals in the field also have a responsibility to use their expertise and experience to make the implications of these choices explicit to policymakers”.

## Supplementary information

Peer Review File
