# Supplementary material for: The geopolitical turn in biotechnology: Implications for Science and Policy
Source: EMBO Rep. 2025 Dec 4;27(1):5–9. doi: 10.1038/s44319-025-00658-4 (PMC12796363; doi:10.1038/s44319-025-00658-4)
Supplement: Supplementary file 1 — Peer Review File [file 44319_2025_658_MOESM1_ESM.pdf]

EMBO reports

The Geopolitical Turn in Biotechnology: Implications for Science and Policy

Ruth Mampuys and Haroon Sheikh

Corresponding author(s): Ruth Mampuys (mampuys@wrr.nl)

Review Timeline:

Submission Date:	31st Oct 25
Editorial Decision:	4th Nov 25
Revision Received:	11th Nov 25
Accepted:	21st Nov 25

Editor: Holger Breithaupt

Transaction Report: This article was editorially reviewed.
